# Identification of tunnels as in potato hydrolases

**DOI:** 10.6026/97320630016021

**Published:** 2020-01-15

**Authors:** Mateusz Banach, Fabian Piotr, Stapor Katarzyna, Konieczny Leszek, Irena Roterman

**Affiliations:** 1 Department of bioinformatics and Telemedicine, Jagiellonian University - Medical College, Lazarza 16, 31-530 Krakow, Poland; 2 Silesian Technical University, Institute of Computer Science, 44-100 Gliwice, Akademicka 16 Poland; 3 Chair of Medical Biochemistry, Jagiellonian University - Medical College 31-034 Kraków Kopernika 7, Poland

**Keywords:** hydrophobicity, tunnels in proteins, submerged active center

## Abstract

Enzymes with an active center hidden in the middle of the molecule in a tunnel-like cavity constitute an interesting object of analysis due to the highly specialized environment for the course of the catalytic reaction. Identifying the tunnel is a challenge in itself. Moreover, the structural conditioning for the course of the reaction provides information on the diversity of the environment, which must necessarily meet the conditions of high specificity. The use of a fuzzy oil drop model to identify residues constituting the walls of the tunnel located in the center of the protein seems highly justified. The fuzzy oil drop model, which assumes the highest concentration of hydrophobicity in the center of the molecule, in these enzymes shows a significant hydrophobicity deficit resulting from the absence of any residues in the central part of the molecule. Comparison of the expected distribution in consistent with the 3D Gaussian distribution where the observed distribution resulting from the interaction of residues in the protein shows significant differences precisely in the positions of residues located near the center of the molecule. The inside characteristics of the tunnel are the background for the enzymatic reaction. This environment additionally constitutes an external force field, which creates favorable conditions for carrying out the catalytic process. The use of fuzzy oil drop model has been verified using the potato (solanum tuberosum) epoxide hydrolase I. This forms the preliminary basis for testing the fuzzy oil drop model. The data presented here provides an impetus for a large scale analysis of all proteins containing tunnels in enzyme structures available in the Protein Data Bank (PDB).

## Background:

The group of enzymes with the so-called submerged active center is the subject of the analysis due to the unexpected location of the active group around the center of the molecule - so from the point of view of the environment it is the center immersed in the body of the protein. An additional element distinguishing this group of enzymes is the presence of a tunnel - an open space on the surface of which catalytic residues are located [1-3]. In the PDBSum database [4], the characteristics of proteins present in this database contain information about the presence of tunnels, giving the composition of the residues constituting the structure of the tunnel surface and the characteristics of those residues. The dimensions of the cavity/tunnel are also given. The tunnels given in the PDBSum database are identified by the MOLE 2.5 program [5-8]. The information given in the PDB Sum database also gives the presence and description of the ligand, if any in the tunnel. In addition to analyzing the phenomenon of location of the active center in such an unusual environment, numerous works provide tools for identifying such enzymes [9]. The subjects of analysis in the context of enzymes with an immersed active center are phenomena associated with evolutionary processes [10]. An additional important problem is the presence of gates whose structural condition is closely related to the regulation of the activity of the enzyme [11]. The subject of the present work is to check the possibility of using the fuzzy oil drop model to identify the tunnel present in the protein molecule. The fuzzy oil drop model assumes the highest concentration of hydrophobicity in the center of the molecule. If a tunnel is present within the protein structure - and therefore free space - then a high mismatch is expected between the expected distribution (3D Gaussian distribution) and the observed distribution, which expresses hydrophobic interactions present in the protein between the residues present in the molecule. Obviously, if free space is present in the central part of the molecule, the observed distribution should show a significant difference in the form of an unexpectedly low level of hydrophobicity in the tunnel environment.

## Materials and Methods:

The comparison of the expected hydrophobicity distribution - in accordance with the distribution expressed in 3D Gaussian distribution spread over the body of the molecule with the distribution present in the analyzed molecule is a criterion for assessing the degree of similarity of these two distributions.

## Sample protein:

The subject of analysis of the present work is potato (solanum tuberosum) epoxide hydrolase, whose structure is available in the Protein Data Bank [12] database under ID 2CJP [13The structural form determined by the Xray technique is a homodimer. The chains are 320 amino acids long. Three ligands are present in the available structure. Their location relative to the tunnel will be determined using a fuzzy oil drop model.

## Description of the fuzzy oil drop model: 

This model has been repeatedly described in other publications [14,15]. Here it is presented in a summary form, enabling the interpretation of the results shown. The model assumes that the idealized distribution of hydrophobicity in the protein (by analogy to the distribution present in the globular micelle) is expressed by means of 3D Gaussian distribution. The size of the ellipsoid is adjusted to the size of the protein molecule using appropriately selected values of the αX, αY and αZ parameters. The highest concentration is expected at the center of the molecule. This concentration decreases as we move away from the center, reaching values close to zero on the surface. This idealized distribution is confronted with the actual distribution resulting from the interaction of residues arranged in a manner specific for each protein molecule. The interaction is determined according to Levitt's function [16]. The magnitude of the hydrophobic interaction depends on the distance between the interacting residues and their own hydrophobicity. The actual observed distribution of hydrophobicity in a particular protein reveals the specificity of each protein revealing areas - parts of the protein showing a locally compatible distribution or locally incompatible with the expected distribution. It should be noted that the hydrophobicity scale can be adopted arbitrarily [15] and that the interaction is calculated for the positions of the so-called effective atoms - the average positions of the atoms contained in the amino acid. The determined distributions after normalization can be compared. The measure of the degree of similarity / differentiation is the value of divergence entropy introduced by Kullback-Leibler [17]. The value thus obtained cannot, however, be interpreted directly. Therefore, a second reference distribution is introduced in the form of a uniform distribution, where all residues represent the same status equal to 1 / N, where N is the number of residues in the chain. This distribution expresses the status without the diversity of the concentration of hydrophobicity at any point in the molecule and thus denies the presence of hydrophobic core. The determined divergence entropy value for the O-T relation (observed against the theoretical one) compared with the divergence entropy value for the O-R relation (where R is a uniform distribution) indicates the O status of the distribution. If O-T> O-R, it means the proximity of the O distribution to the R distribution, and the protein structure is interpreted as lacking the presence of the hydrophobic core. Otherwise, the protein is treated as folded according to the structure of the micelles and having a hydrophobic core. To eliminate the use of two values, the RD (Relative Distance) parameter was introduced expressing the ratio of the O-T measure to the sum of the O-T and O-R measures. A value of RD <0.5 indicates the presence of hydrophobic core. The analysis described above can be performed for any selected section of the chain by identifying its status. Such analysis requires previous normalization of Ti and Oi values for a selected chain fragment. The analysis of the discussed hydrolase was based on the fuzzy oil drop model identifying the deviation of the observed distribution (O) from the expected (T) of the entire protein molecule, segments showing deviations and identifying the causes of the identified discrepancy.

## Results and Discussion:

### Status of the complete molecule of the discussed hydrolases: 

**Figure 1:**
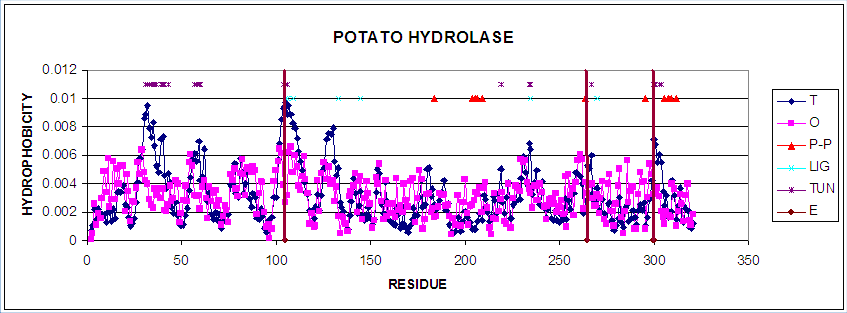
Profiles T (theoretical) and O (observed) hydrophobicity as it appears in the chain A. P-P - residues engaged in protein-protein interaction, Lig - residues engaged in ligand binding, TUN - denotes residues localised on the surface of the tunnel, E - catalytic residues (vertical lines)

The molecule of this hydrolase is a single-domain globular structure containing a centrally located Beta plate in a parallel / antiparallel system. The molecule also contains 20 sections of helical structure. There are no disulphides in the molecule. The status determined by the RD parameter is 0.545. This means that the whole molecule does not show an ordered hydrophobic nucleus within the meaning of the fuzzy oil drop model. Visualization of these distributions is shown in Figure 1.

The residues representing significantly low hydrophobicity level in respect to the expected one are assumed to be localized on the tunnel surface. The profile has sections that express the expected hydrophobic center: 24-48, 51-64, 97-118, 121-137 and 296-309. The graphs also show significantly lower levels of hydrophobicity observed for these sections. In order to search for fragments of the chain with the distribution as expected from the analysis, those residues that show the highest differences were eliminated.

### Tunnel identification: 

**Figure 2:**
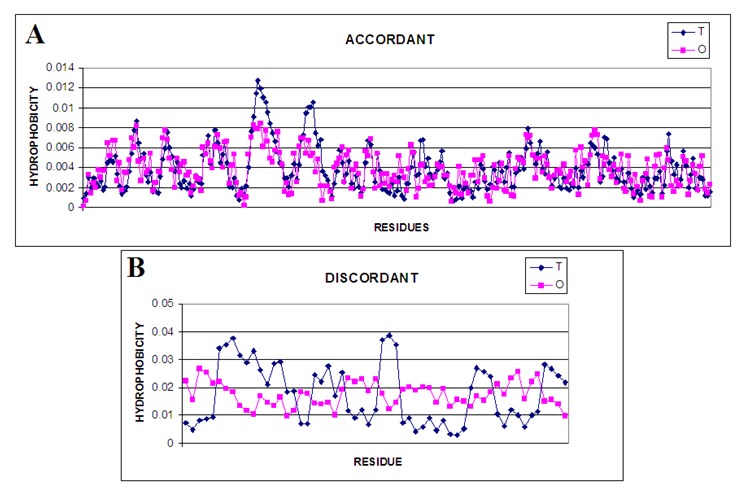
T and O profiles for parts of the chain: A - selected residues showing consistent levels of Ti and Oi B - selected residues identified as showing significantly different levels.

In order to identify residues whose status shows a deviation from the expected distribution, the absolute values of Ti and Oi differences were calculated. Elimination of these residues from divergence entropy calculations and counting the status of the set of eliminated residues (divergent status) resulted in a decrease of the RD value for the part without these residues to the level of 0.386, while the residues showing significant differences determined by the value of RD = 0.912.The respective profiles are shown in Figure 2. Numbers of residues are in the position ordered however the eliminated residues are omitted. The positions of residues can be recognized using the Figure 1.

In Figure 2B, in addition, segments showing a local deficiency in hydrophobicity observed relative to the expected can be identified. The status of these residues reveals their central location (high Ti values) at much lower levels of observed hydrophobicity. It can be assumed that these residues form the inner surface of the tunnel. Residues exhibiting a level of Oi higher than Ti are those positions that exhibit high hydrophobicity on the surface (as evidenced by low Ti). These residues are probably involved in the complexation of the second molecule (the structure of the protein in question in PDB is a homodimer).

**Figure 4:**
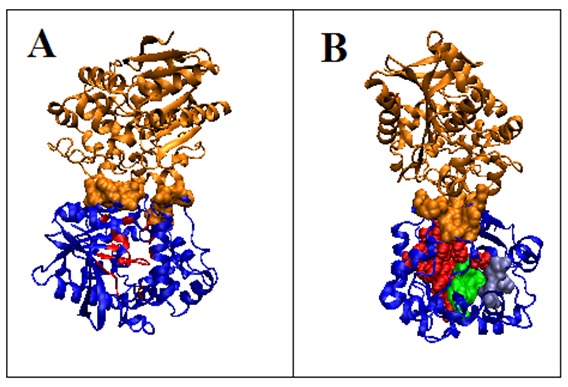
3D struktura potato (solanum tuberosum) epoxide hydrolase i. Chain A - blue, Chain B - orange A - residues on the tunnel surface - red, orange space filling - residues in chain A engaged in the complexation of chain B. B - residues on the tunnel surface tunnel engaged in ligand complexation - green space filling, residues not engaged in tunnel surface engaged in ligand complexation - light blue

The residues shown in Figure 2B representing higher hydrophobicity level that the expected one appear to be engaged in protein-protein interaction. The residues indicated in PDBSum as involved in ligand binding appear to be those that constitute the tunnel wall. These are residues: 105, 106, and 235 and in close proximity to the tunnel surface: 270 and 109. Figure 4B shows the positions of these residues in the spatial structure.

## Conclusions:

The presence of immersed enzymatic centers identified on the basis of the Voronoi diagram [5] using geometry appears to have competition in the form of a fuzzy oil drop model. The identification of tunnels in proteins is based on the identification of differences in the level of hydrophobicity expected from experimental observation. Moreover, knowledge of the residues involved in complexing another protein molecule as well as the status of residues involved in ligand complexing versus tunnel status helps to identify the involvement of tunnel-covering residues in ligand interactions. Further, recognition of the characteristics of catalytic residues allows determining the availability of these residues for substrates. The external force field for catalytic reaction can also be identified using the fuzzy oil drop model. Visual inspection of the protein under consideration shows the significant deficiency of hydrophobicity yet only on one site of the tunnel. It implies the role of differentiated characteristics of the tunnel walls. Positive evaluation of the fuzzy oil drop model for identifying tunnels in protein molecules suggests checking the effectiveness of using this model for a larger number of proteins with current tunnels [11]. We propose to test such hypothesis in future investigations.

## Figures and Tables

**Figure 3:**
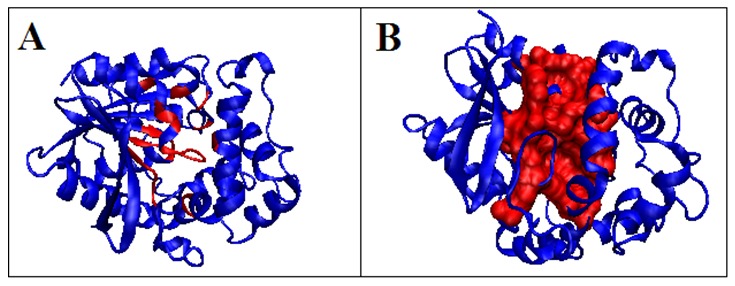
3D presentation of the discussed hydrolase structure with highlighted residues exhibiting a local hydrophobicity deficit. A - residues on tunnels surface – red; B - residues on tunnels surface - red space filling
